# Mechanism of Action of IL-7 and Its Potential Applications and Limitations in Cancer Immunotherapy

**DOI:** 10.3390/ijms160510267

**Published:** 2015-05-06

**Authors:** Jianbao Gao, Lintao Zhao, Yisong Y. Wan, Bo Zhu

**Affiliations:** 1 Institute of Cancer, Xinqiao Hospital, Third Military Medical University, Chongqing 400037, China; E-Mails: nwgjb@163.com (J.G.); chaoren72@126.com (L.Z.); 2 Lineberger Comprehensive Cancer Center, University of North Carolina at Chapel Hill, Chapel Hill, NC 27599, USA; 3 Biomedical Analysis Center, Third Military Medical University, Chongqing 400038, China

**Keywords:** IL-7, cancer, immunotherapy

## Abstract

Interleukin-7 (IL-7) is a non-hematopoietic cell-derived cytokine with a central role in the adaptive immune system. It promotes lymphocyte development in the thymus and maintains survival of naive and memory T cell homeostasis in the periphery. Moreover, it is important for the organogenesis of lymph nodes (LN) and for the maintenance of activated T cells recruited into the secondary lymphoid organs (SLOs). The immune capacity of cancer patients is suppressed that is characterized by lower T cell counts, less effector immune cells infiltration, higher levels of exhausted effector cells and higher levels of immunosuppressive cytokines, such as transforming growth factor β (TGF-β). Recombinant human IL-7 (rhIL-7) is an ideal solution for the immune reconstitution of lymphopenia patients by promoting peripheral T cell expansion. Furthermore, it can antagonize the immunosuppressive network. In animal models, IL-7 has been proven to prolong the survival of tumor-bearing hosts. In this review, we will focus on the mechanism of action and applications of IL-7 in cancer immunotherapy and the potential restrictions for its usage.

## 1. Introduction

Interleukin-7 (IL-7) is a cytokine essential for the adaptive immune system. T lymphopoiesis in the thymus has been shown to be highly IL-7 dependent in mice [1]. In addition, IL-7 is important for T cell homeostasis and lymphopenia-driven proliferation [2]. It regulates lymph nodes (LN) organogenesis by controlling the pool of lymphoid tissue inducer (LTi) cells. By activating several intracellular signal pathways, IL-7 promotes the cell survival and proliferation of both naïve and memory T cells [3]. For its function on the maintenance of the naïve T cell pool and the survival of memory T cells, several clinical trials have been designed to use IL-7 to enhance immunity to viral infections and have achieved some success.

Cancer is a common disease worldwide and causes thousands of deaths annually. One important reason for tumor progression lies in that tumors can suppress the normal immunological surveillance function of the immune system of the organism to avoid detection [4]. Cancer patients often have an immunosuppressive status with lower T cell counts. In the tumor microenvironment, less infiltrated effector immune cells are predominantly composed of exhausted cytotoxic T lymphocytes (CTLs), T helper cell type2 (Th2) and macrophages (M2), which are tolerant toward tumors. There are also more T regulatory cells (Treg) and myeloid-derived suppression cells (MDCS) to inhibit effector immune responses.

In addition to killing cancer cells by chemotherapy and radiotherapy, immunotherapy has emerged as an attractive area of interest in recent years, which utilizes the immune system of patients themselves. IL-7 is an ideal target used to enhance the function of the immune system. It can reconstitute the immune system [5,6], improve T cell function *in vivo* and antagonize the immunosuppressive network. Thus, we focused on the application of IL-7 as an immunotherapy for cancer treatment and the action mechanism of IL-7. We also discuss limitations for the use of IL-7.

## 2. Biology of IL-7 and Its Signaling

IL-7 is mainly produced by non-hematopoietic cells including keratinocytes in the skin, fibroblastic stromal cells in the bone marrow [7] and lymphoid organs [8,9], epithelial cells in the thymus [7], prostatic epithelium [10] and the intestine. Immune cells, such as dendritic cells (DCs) can also produce IL-7 [11]. Moreover, IL-7 transcripts and proteins have also been found in normal adult human hepatic tissue produced by cells of lymphoid morphology [12,13]. The human IL-7 gene locus is 72 kb in length, resides on chromosome 8q12-13 and encodes for a protein of 177 amino acids with a molecular weight of 20 kDa. While the murine IL-7 gene is 41 kb in length, it encodes a 154 amino acids protein with a molecular weight of 18 kDa [14].

The receptor of IL-7 is a heterodimer that consists of two chains: IL-7Rα (CD127), which is shared with thymic stromal lymphopoietin (TSLP), and the common γ chain (CD132) for IL-2, IL-4, IL-9, IL-15 and IL-21. The γ chain is expressed on all hematopoietic cell types, while IL-7Rα is mainly expressed by lymphocytes, including common T/B lymphoid precursors, developing T and B cells, naïve T cells and memory T cells [14]. Innate lymphoid cells (ILCs) are critical in lymphoid organ development and innate immune responses to pathogens. IL-7Rα is also found in ILCs, such as NK cells and gut-associated lymphoid tissue (GALT)-derived LTi cells. IL-7 can also regulate lymphoid organogenesis by controlling the pool of LTi cells [15]. IL-7Rα is regulated by stimulative transcription factors, GABPα and Foxo1 as well as inhibitory Gfi-1 [16,17,18,19]. TGF-β promotes IL-7Rα expression via the inhibition of Gfi-1 expression [20]. There is another type of IL-7 receptor: soluble IL-7R, which competes with cell-associated IL-7R to reduce excessive IL-7 consumption by IL-7R expressing target cells and enhances the bioactivity of IL-7 when the cytokine is limited [21].

There are two main signaling pathways responsible for the function of IL-7: Jak-Stat and PI3K-Akt [3]. IL-7Rα is associated with the protein tyrosine kinase Janus kinase 1 (Jak1), and the cytosolic tail of the γ chain is associated with Jak3. Binding of IL-7 to its receptor causes activation of Jaks in the cytosol, phosphorylating signal transducer and activator of transcription (STAT) proteins. The dimeric phosphorylated STAT (pSTAT) proteins subsequently translocate into the nucleus to activate gene expression. Via the Jak3-Stat5 pathway, IL-7 activates the anti-apoptotic genes, Bcl-2 and Mcl-1, and suppresses pro-apoptotic proteins, such as Bax and Bak. Consequently, naïve and memory T cells survive. This function is dose-dependent, such that a higher concentration of IL-7 induces thymic emigrant T cell proliferation, while lower concentrations sustain cell survival [22]. By activating the PI3K-Akt pathway, IL-7 downregulates the cell cycle inhibitor p27^kip1^ to induce the expression of cyclin D1 for cell cycle progression [23]. Moreover, it promotes glucose transporter 1 expression, glucose uptake and mitochondrial integrity to positively regulate cell metabolism and size [14,24].

## 3. Function of IL-7 in T Cell-Mediated Immune Response and the Underlying Mechanisms

The immune system protecting the organism from cancer relies on the size of its T lymphocyte pool, especially the CD8^+^ T cell pool. This pool is maintained in a dynamic balance. Antigen-specific effector T cells fulfill their mission and subsequently die. Then expansion of new T cells supplements this pool. IL-7 retains this balance in three ways: thymopoiesis, homeostasis proliferation and life-support.

### 3.1. IL-7 Signaling Is Essential for Thymopoiesis

T lymphocytes are mainly generated in the thymus where IL-7 is necessary for their development. Mice deficient in IL-7^−/−^, IL-7Rα^−/−^, γc^−/−^ or Jak3^−/−^ suffer from severe lymphopenia, particularly for T and NK cells. A human mutation in IL-7Rα results in a syndrome of severe combined immunodeficiency (SCID) with an absence of T cells [1]. During lymphoid cell development, the expression of IL-7Rα is tightly controlled [5]. The development of αβ T-cells in the thymus includes several stages, *i.e.*, double-negative (DN) stages (DN1–4), immature single positive (ISP), double-positive (DP) and single positive (SP) CD4^+^ or CD8^+^ T cells. Early thymocyte progenitors do not express IL-7Rα. As T cell lymphopoiesis progresses, DN2 progenitors have the highest IL-7Rα expression while DN4 cells express the lowest levels of IL-7α. IL-7 activates the transcription factor NFATc1 in DN thymocytes by phosphorylating Tyr371 in the regulatory region of NFATc1, a pathway that is critical for the survival and development of DN thymocytes [25]. Furthermore, DP cells lose their expression of IL-7Rα, but SP cells re-express it where IL-7 signaling promotes their survival and proliferation [26]. IL-7 signaling is also important for γδ T-cells in inducing γ-chain rearrangement by enhancing histone acetylation and locus accessibility [27].

### 3.2. IL-7 for Peripheral Homeostasis of T Cells

IL-7 plays a critical role in peripheral T cell homeostasis [2]. There are very limited amounts of IL-7 under physiological conditions *in vivo*. Stromal cells produce IL-7 at relatively constant amounts and independently of external stimuli. Regulation of the function of IL-7 relies on IL-7Rα levels [26]. In addition, binding of IL-7 downregulates IL-7Rα expression by decreasing its gene transcription [19]. IL-7Rα is highly expressed on naive and central memory T cells. Once primed by the antigen, naïve T cells differentiate into effector T cells and lose IL-7Rα expression in an altruistic manner such that more naïve T cells obtain limited IL-7 to survive and proliferate. Nevertheless, IL-7Rα is re-expressed when effector T cells differentiate to the memory stage.

In human lymphopenia diseases, such as HIV infection, idiopathic CD4^+^ T lymphopenia, high dose chemotherapy and auto-immune diseases, patients often have increased circulating levels of IL-7 and a strong inverse correlation between the levels of IL-7 and the number of CD4^+^ T cells. Higher levels of IL-7 induce T cell homeostasis proliferation outside of the thymus. After the recovery of CD4^+^ T cell populations, IL-7 returns to homeostatic levels due to naive CD4^+^ T cells expression of IL-7R, which consumes IL-7 to maintain their survival [28]. In lymphopenic mice, homeostatic T cell proliferation requires the transduction of signals from T cell antigen receptor (TCR) interactions with self-MHC class II and class I ligands, as well as with IL-7R [29]. However, IL-7 has more potent effects on CD8^+^ T cells compared with CD4^+^ T cells. In addition, CD8^+^ T cells survive better and proliferate faster than CD4^+^ T cells. Furthermore, IL-7 signaling on IL-7Rα^+^ DCs downregulates the expression of MHC class II on DCs, which is critical for the expansion of CD4^+^ T cells [11]. During CD8^+^ T cell homeostasis, IL-7 signaling must be intermittent due to continuous IL-7 signaling results in increased pSTAT5, which can activate IFN-γ gene expression [30] and cell apoptosis triggered by IFN-γ [31]. The intermittent interruption of IL-7R signaling is induced by TCR stimulation, which is dependent on the calcium-sensitive protease calpain to cleave the cytosolic tail of the γ chain and dissociate Jak3 from IL-7R [32]. The TCR signal also acutely reduces Jak1 protein levels and prevents its synthesis to impair IL-7 signaling via microRNA-17 [33].

### 3.3. IL-7 in SLOs Is Important for Adaptive Immune Responses

Adaptive immune responses against tumors are generated in secondary lymphoid organs (SLOs), particularly in LN draining of pathological sites. They provide suitable microenvironments for the activation and expansion of antigen-specific lymphocytes. In these collaborative environments, lymphocytes move along stromal networks to scan the surfaces of antigen-presenting cells (APCs) for cognate antigens. The interactions between stromal and hematopoietic cells in SLOs are critical for the function of immune cells [34]. There are different stromal subtypes according to their surface marker: CD31 and podoplanin (PDPN, also known as gp38 and T1α) [35]. Fibroblastic reticular cells (FRCs, gp38^+^CD31^−^) in SLOs secrete chemokines CCL19 and CCL21, to attract T cells and DCs and orchestrate a complex network of collagens and extracellular matrix to facilitate the migration of these cells [36,37,38]. Simultaneously, FRCs are the main cellular source of IL-7 in the periphery, which is a survival signal to lymphocytes immigrating into SLOs [8]. The lymphatic vessel is enclosed with lymphatic endothelial cells (LECs, gp38^+^CD31^+^), which not only convey signaling in the lymph further upstream, but it also induces peripheral tolerance [35,39,40]. LECs are the prominent source of IL-7 in human fetal mesenteric LNs [9]. Our team recently found that IL-7 produced in the spleen of tumor-bearing mice was significantly decreased (data not published). Inadequate supply of IL-7 in SLOs might be insufficient to support the survival of activated T cells, thereby aggravating the immunosuppression of cancer.

## 4. Application of IL-7 in Cancer Immunotherapy

Cytokines have been applied in cancer immunotherapy for many years, of which IL-2 was approved by the U.S. Food and Drug Administration (FDA) for the treatment of patients with metastatic melanoma. But it required high-dose of IL-2 to achieve significant clinical response which resulted in dose-related fever, serious biochemical abnormalities in the liver and kidney, and capillary leak [41]. IL-2 stimulates proliferation of effector cells that kill cancer cells but it also plays an important role in the maintenance of regulatory T (Treg) in the periphery that suppresses the immune response [42]. IL-15 has a pivotal role in life and death of NK and memory CD8^+^ T cells [43]. But it will cause fever, rigors and blood pressure dropping [42]. It also induces the expression of the immunosuppressive receptor, programmed cell death protein 1 (PD-1) and increases the immunosuppressive cytokine IL-10 secreted by CD8^+^ T cells [44].

Investigators aim to utilize IL-7 to enhance the efficacy of tumor regression. A set of preclinical trials has proven that IL-7 can prolong the survival of tumor-bearing hosts in several tumor models. In murine mammary carcinoma, the administration of IL-7 and IL-15 after radiofrequency thermal ablation (RFA) resulted in a relapse-free survival and showed inhibition of metastatic nodules in their lungs [45]. Adoptive immunotherapy, wherein tumor-specific CTLs are activated and expanded *in vitro* with bryostatin and ionomycin, that were infused back into the body of the animals, has greater regression of established melanoma and 4T1 mammary carcinomas *in vivo* if the cells were cultured in IL-7 and IL-15 compared with IL-2 alone [46,47]. Interestingly, intratumoral injection with adenoviral IL-7 transduced DCs resulted in complete tumor regression in a murine lung cancer model [48]. IL-7 producing whole cell vaccine is also proved to evoke an effective anti-cancer immune response, probably mediated by NK1.1^+^ cells for subcutaneous prostate cancer challenge [49], while the GM-CSF producing vaccine is good at intraprostatic tumor challenge [50]. In the clinical trials reported in 2006, 2008 and 2010, patients with different kinds of cancers such as metastatic melanoma or sarcoma were injected subcutaneously with different doses of IL-7. Little toxicity was seen except for transient fevers and mild erythema. Circulating levels of both CD4^+^ and CD8^+^ T cells increased significantly and the number of Treg reduced. TCR repertoire diversity increased after IL-7 therapy. But the anti-tumor activity of IL-7 was not well evaluated [51,52,53].

### 4.1. IL-7 for Immune Reconstitution in Cancer Patients

Cancer patients often suffer from immunosuppression with low T cell counts [54]. This situation will deteriorate when the patients received chemotherapy as a side-effect of drugs via myelosuppression. IL-7 administration can enhance their immune reconstitution [5,6], mainly from the homeostatic expansion of peripheral T cell populations. IL-7 therapy not only increases both T cell counts [55] and the diversity of T cells recognizing different antigens [53], but it also promotes the homing of T cells to lymphoid tissues where antigen recognition occurs [56]. It has also been reported that IL-7 can increase thymopoiesis to fill-up the pool of naïve T cells [57]. RhIL-7 has been applied in a phase I study with a significant increase in peripheral CD4^+^ and CD8^+^ T lymphocytes in patients with refractory malignancy [51].

### 4.2. IL-7 Enhances the Function of Effector Immune Cells

The IL-7 treatment can improve the function of immune cells *in vivo*. As an adjuvant, IL-7 enhances long-term tumor antigen-specific CTLs responses in both quantity and quality after immunization with recombinant lentivector [58]. First, it can recruit multiple effector cells, such as CTLs, natural killer (NK) and NKT cells to infiltrate the tumor sites. The T helper type 17 (Th17) subset of CD4^+^ T cells is well known in promoting inflammation and pathology in animal models. In addition to CTLs, the number of infiltrated Th17 cells also increases with IL-7 adjuvant [59], which is potentially driven by the increased total number of CD4^+^ T cells. Next, it represses Casitas B-lineage lymphoma-b (Cbl-b), a negative regulator of T cell activation, to maintain CD8^+^ T cells survival [59]. These CD8^+^ T cells can continue to proliferate even under conditions where the antigen and other cytokines are limited. Simultaneously, increased CTLs infiltration produces more IFN-γ in mouse melanoma, colon and breast cancer models [60,61,62] and upregulates granzyme B expression [59]. Memory T cells can persist long-term to patrol the entire organism for residual tumor cells. IL-7 can also improve the survival of IL-7-Rα^+^ central memory populations and promote the production of IL-7Rα^+^ long-living memory stem T cells (TSCM), which can self-renew and demonstrates the plasticity to differentiate into effectors [63]. The antigen recognition relies on the host APC acting with TCR. The utility of the chimeric homeostatic cytokine, IL-7/IL-7Rα-Fc, partly restored APC activities in lung cancer, with a stronger capacity to process and present antigens to activate CD8^+^ T cells [64].

### 4.3. IL-7 Antagonizes the Immunosuppressive Network

Several reports have shown that IL-7 is able to antagonize the immunosuppressive network [59]. This network consists of inhibitory cytokines and immunosuppressive cells, such as Treg and MDSC. It is well established that cancer-associated CD8^+^ T cells exhibit a phenotype known as exhaustion, with high PD-1 expression. IL-7 can diminish PD-1 expression on activated CD8^+^ T cells such that they are revitalized [59]. Some tumor cells can secrete TGF-β that signals via the SMAD protein to inhibit the proliferation of CD8^+^ T cells. IL-7 can abrogate this inhibitory effect via an induction of the expression of SMAD ubiquitylation regulatory factor 2 (SMURF2) [59]. Treg accumulates in the tumor microenvironment and inhibits immune responses. Treg cells have low expression of CD127 and high level of CD132 on their surface. IL-7 can directly abrogate the Treg-mediated suppression of effector T cell proliferation [65] and decrease the population of Treg in spleen of lung cancer model [66]. Intralesional injection with IL-7 and IL-15 in RFA-treated animals can also reduce the proportion of MDSC present in the spleen in murine mammary carcinoma [45]. However, IL-7 has little effect on the proliferation of Treg and MDSC. Thus, the decreased proportion of Treg and MDSC may be the result of significant proliferation of naïve T cells with IL-7 therapy.

## 5. Potential Caveat of the Use of IL-7

IL-7 signaling is well known to occur in either the initiation or maintenance of some lymphocyte-derived tumors, such as T-cell acute lymphoblastic leukemia [67,68]. IL-7 appears to be involved in autocrine circuitries to maintain the growth of lymphoma cells [69]. Lesional skin from cutaneous T-cell lymphomas patients can secrete more IL-7, which contributes with the proliferation of lymphoma cells [70]. Nevertheless, both IL-7 and its receptor are also found in some solid tumor samples. IL-7 mRNA is detected in colorectal, esophageal, renal, head and neck squamous cell carcinoma, as well as in Warthin’s tumors of the parotid gland [71]. Prostatic epithelia constitutively produce IL-7 but decrease its production dramatically during the neoplastic glands formation [10]. But serum titers of IL-7 significantly increased in prostate cancer patients [72,73,74]. IL-7 levels were strongly associated with ovarian cancer and could be used in combination with CA-125 to distinguish between malignant and benign ovarian tumors [75]. In non-small-cell lung carcinoma patients’ biopsies, IL-7 was highly expressed by bone invading cells [76]. IL-7R mRNA is expressed in the breast, lung, colon, renal and CNS cancer cell lines [77]. High expression of tumor IL-7R is associated with worse outcome in patients with stage I lung adenocarcinoma [78].

There is no data demonstrating the exact cell source of IL-7 detected in tumor sample. Was it obtained from tumor cells themselves or from stromal cells in the tumor tissue? Moreover, the source of increased IL-7 in serum was not defined to-date. It is unclear whether IL-7 may signal tumors themselves to accelerate the progression of cancer. There has not been good data to demonstrate that IL-7 receptor expressed on solid tumors is functional or that IL-7 contributes to tumor growth [79]. As a result, further research studies are needed to justify the function status of IL-7R expressed on tumor cells. Perhaps different tumor models will have a different reaction to IL-7 therapy. IL-7 is likely to work only in some types of cancer.

## 6. Conclusions

**Figure 1:**
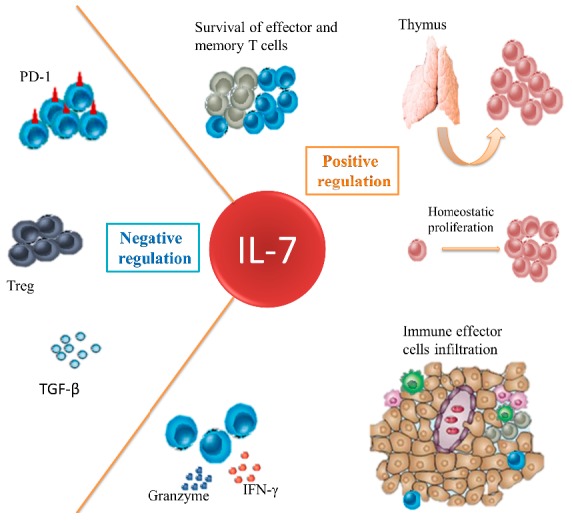
Effects of IL-7 in cancer immunotherapy. IL-7 can maintain the survival of CTLs and memory T cells. It promotes immune reconstitution both from the thymus-independent homeostatic expansion of peripheral T cells and thymopoiesis. It enhances the efficacy of tumor regression by enhancing effector cells infiltration, such as CTL, NK, NKT and Th17 with an upregulated killing capability. It also antagonizes the immunosuppressive network.

Modulation of the IL-7 axis is an ideal tool for repairing damaged immune systems in cancer patients, particularly those who received chemotherapy (Figure 1). IL-7 can promote immune reconstitution both from thymus-independent homeostatic expansion of peripheral T cells and thymopoiesis. It guides more CTLs and other immune effector cells infiltration with better survival and upregulated killing activities. IL-7 fights against the immunosuppressive network to improve immune function on cancer cells. Blockade of IL-7 and/or its receptor is feasible in lymphoid malignancies that are dependent upon IL-7 signaling. However, the function of IL-7 and its receptor expressed in some solid tumor remain to be clarified. The situation will deteriorate if the solid tumor expresses a functional IL-7R that promotes cancer progression. Whether IL-7R should be detected in tumor sample before IL-7 administration will require further research.
